# Intracardiac Metastasis From Non-Small Cell Lung Cancer

**DOI:** 10.3389/fonc.2015.00168

**Published:** 2015-07-21

**Authors:** Vivek Verma, Geoffrey A. Talmon, Weining K. Zhen

**Affiliations:** ^1^Department of Radiation Oncology, University of Nebraska Medical Center, Omaha, NE, USA; ^2^Department of Pathology, University of Nebraska Medical Center, Omaha, NE, USA

**Keywords:** non-small cell lung cancer, metastases, heart failure, radiotherapy, chemotherapy

## Abstract

A 56-year-old female with history of stage IIA adenosquamous lung carcinoma treated 13 months prior to presentation with lobectomy, mediastinal lymph node dissection, and adjuvant chemotherapy, presented for several weeks of worsening dyspnea. Exam was non-focal aside from tachycardia. Computed tomography of the chest revealed a large 4 cm × 5 cm mass in the bilateral ventricular myocardium. There was also evidence of metastatic disease elsewhere in the body, including a supraclavicular lymph node that was positive for metastatic adenosquamous lung carcinoma. She started whole heart radiotherapy and was to commence chemotherapy but passed away. This report discusses important aspects of diagnosis of this not uncommon condition that many oncologists may come across. We also discuss differential diagnosis of an isolated intracardiac mass as first-diagnosis presentations, and discuss the great importance of multidisciplinary cardio-oncologic management and clinical prioritization.

## Introduction

A 56-year-old woman presented for several weeks of worsening dyspnea on exertion and non-productive cough. She had a history of stage IIA adenosquamous carcinoma of the lung (left lower lobe) treated 13 months ago with lobectomy and mediastinal lymph node dissection and adjuvant chemotherapy (carboplatin/paclitaxel and subsequently pemetrexed), with no evidence of disease on multiple subsequent computed tomography (CT) scans. After her initial diagnosis, she had ceased smoking and had previously overall felt well. Her weight had been stable and had no pain other than left-sided chest pain when coughing. She also endorsed night sweats and palpitations.

On examination, lungs were clear to auscultation bilaterally and cardiac exam was unremarkable except for mild tachycardia. The remainder of the examination was normal with exception of a firm but mobile 1 cm lymph node palpated in the left supraclavicular fossa.

**Figure 1:**
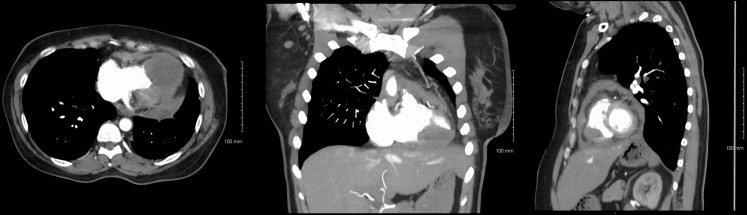
**Axial (left), coronal (center), and sagittal (right) CT with contrast images showing intracardiac metastasis**.

On presentation, CT chest with contrast (Figure 1) demonstrated normal postoperative changes of left lower lobectomy. There was a large intracardiac mass, 4 cm × 5 cm, involving bilateral ventricular myocardium. Also present was left-sided supraclavicular lymphadenopathy and irregular pericardial thickening suggestive of pericardial tumor involvement.

**Figure 2:**
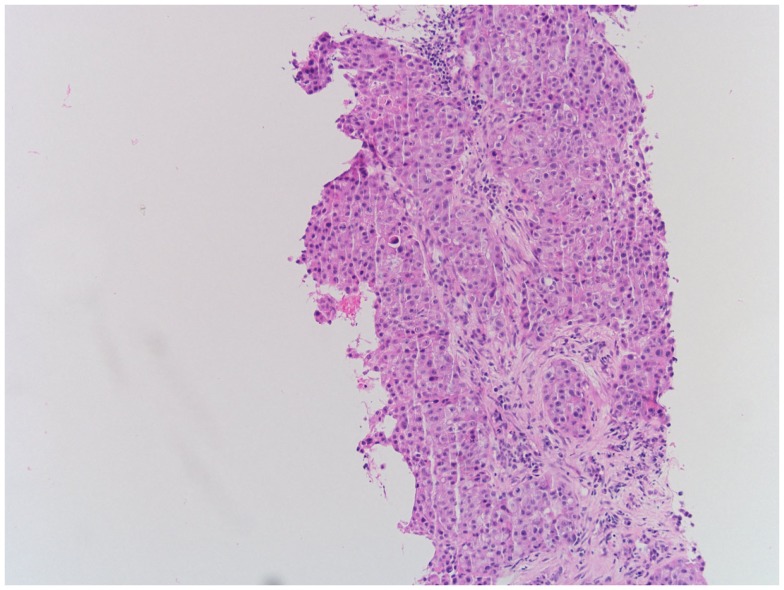
**Hematoxylin-eosin staining of left supraclavicular lymph node demonstrating recurrent NSCLC**.

Biopsy of the left supraclavicular lymph node revealed a metastasis of the patient’s non-small cell lung cancer (NSCLC), as depicted in Figure 2. Thus, though the cardiac mass was not biopsied, its radiological appearance (1) as well as relatively quick growth to 4 × 5 cm size in 13 months was concerning for tumor recurrence.

She was to have port placement for chemotherapy, and started whole heart radiotherapy at a dose of 3,000 cGy in 12 fractions of 250 cGy each. Unfortunately, this patient completed a portion of radiotherapy (1,750 cGy) and nearly 3 weeks after diagnosis, was to undergo port placement for systemic therapy (nivolumab) but passed away from supraventricular tachycardia, which progressed to pulseless cardiac arrest.

## Background

Cardiac metastases, most commonly from lung cancer (30–40%), are not as uncommon as expected, with incidences up to 18% in the literature; the average incidence around 10% of all patients with malignancies seems less in clinical practice, likely due to most metastases having been discovered incidentally and smaller in size (2).

There have been several case reports of intracardiac metastases from various types of cancers, including lung cancer as in the present case (3). As with this patient, metastases can be symptomatic, or more commonly, asymptomatic (4). Though diagnosis is usually by echocardiography, incidental discoveries have been shown with other modalities, such as endobronchial ultrasound (5). Tumors can be located in any atrial or ventricular chamber, or possibly even multiple tumor foci within a single chamber at a time (6). Treatment is very variable from study to study, and thus is quite controversial. It is usually done on a case-by-case basis, and treatments have ranged from surgical resection (7), chemotherapy (8), and even primary radiotherapy (9).

## Discussion

Though tumor recurrence in this patient was the most likely diagnosis, first-time diagnoses in similar patients provide a discussion-worthy differential diagnosis. Tumor thrombus is an important item on the differential diagnosis, also because coagulopathy of malignancy can present in such a manner and would result in entirely different management including anticoagulation (10). Cardiac abscesses can get large and also be associated with regional lymphadenopathy, but are usually non-homogeneous on imaging with non-erosive features, and are far less likely than tumor recurrences. Lymphoma is the second most common cause of metastatic cardiac tumors, and could be likely given concurrent lymphadenopathy in other areas, but a 13-month interval may not be an optimal timing of developing secondary cancer, and new primary lymphoma would not be the most common cause as much as an NSCLC recurrence (11). Biopsy confirmed that recurrence was from the NSCLC and not lymphoma. Other items on the differential diagnosis that are important to consider are second primary lung cancer or a local recurrence that extrinsically invades the heart, and less likely primary heart tumors, due to differences in management accordingly.

Initial workup in such patients is hence very important, because chest imaging may not even be performed at many centers if symptoms can be easily explained by the vastly more common exacerbations of congestive heart failure (CHF) and/or chronic obstructive pulmonary disease (COPD). Indeed, many patients with lung cancer history also have comorbidities including CHF and COPD for which symptomatic treatment could delay or prevent diagnosis of an intracardiac mass if there were no other findings on physical examination (such as supraclavicular lymphadenopathy in this patient). With history of the more aggressive adenosquamous lung cancer, however, greater diagnostic suspicion is certainly warranted (12).

Because intracardiac metastases can be hemodynamically threatening, management is important for clinicians to be aware of; multidisciplinary management by a team of medical oncologists, cardiologists, radiation oncologists, and pulmonologists is crucial. Maintenance of hemodynamic stability is of greatest concern; this patient had severely reduced ventricular systolic function. As a result, this patient’s tachycardia was likely compensatory; thus, beta-blockers make the clinical condition worse, so medication modification is also important.

Treatment options must also be handled by a multidisciplinary team. Radiotherapy is efficacious for quick cell killing, so whole heart radiotherapy was recommended. Whole heart radiotherapy is the most common form of radiotherapy existent in the literature, including fraction sizes ranging mostly from 200 to 300 cGy (discussed subsequently). Chemotherapy (e.g., carboplatin/paclitaxel) versus newer biologic agents, such as the PD-1 inhibiting antibody nivolumab, which has shown efficacy in previously treated and advanced NSCLC, should also be considered (13).

Prognosis for patients with intracardiac metastases is correspondingly limited, with limited clinical experience. It is reasonable to conclude that prognosis for patients with intracardiac metastases is poor, likely because the disease is metastatic to begin with. A suggestive and relatively large study from Taiwan (14) retrospectively analyzed 48 patients with intracardiac metastases from hepatocellular carcinoma (HCC) and compared outcomes with age-, stage-, and gender-matched HCC patients. The median survival was not different (*p* = 0.67) between those with intracardiac metastases (102 days) and the HCC patients without intracardiac metastases (68 days). This suggests that metastatic cancer is likely the cause of death in these patients, regardless of whether intracardiac involvement is present, provided acute aberrant hemodynamics is stabilized. However, it is known from another retrospective report of 11 patients that heart radiotherapy can potentially improve survival (15). Though not statistically analyzed, radiotherapy (of varied doses, 25–60 Gy) improved median survival to 10.5 months as compared with 3.5 months without radiotherapy. Thus, with more clinical experience, it will also be important to better delineate radiotherapy doses in these patients, for which there are no standard guidelines at present.

## Concluding Remarks

In summary, intracardiac metastases are not uncommon and are serious complications of many tumors, most commonly lung cancers. Diagnosis can often be difficult if imaging is not obtained, so clinical suspicion needs to be exercised especially with a primary tumor with poor prognostic factors. Multidisciplinary cardio-oncologic management with prioritization on cardiovascular stability and rapid tumor killing for symptomatic cardiovascular benefit is warranted, as are systemic therapy options for likely other sites of involvement.

## Consents and Approvals

Institutional Review Board approval is not required for single-patient cases at the University of Nebraska Medical Center. Consent was obtained from the patient’s next of kin for this report.

## Conflict of Interest Statement

The authors declare that the research was conducted in the absence of any commercial or financial relationships that could be construed as a potential conflict of interest.
